# Acyl-CoA-binding protein (ACBP): a phylogenetically conserved appetite stimulator

**DOI:** 10.1038/s41419-019-2205-x

**Published:** 2020-01-06

**Authors:** Nikolaos Charmpilas, Christoph Ruckenstuhl, Valentina Sica, Sabrina Büttner, Lukas Habernig, Silvia Dichtinger, Frank Madeo, Nektarios Tavernarakis, José M. Bravo-San Pedro, Guido Kroemer

**Affiliations:** 1 Institute of Molecular Biology and Biotechnology, Foundation for Research and Technology - Hellas, Nikolaou Plastira 100, 70013 Heraklion, Crete Greece; 2 Department of Biology, University of Crete, 70013 Heraklion, Crete Greece; 3 Institute of Molecular Biosciences, NAWI Graz, University of Graz, Humboldtstrasse 50, 8010 Graz, Austria; 4 Metabolomics and Cell Biology Platforms, Gustave Roussy Cancer Campus, Villejuif, France; 5 Inserm U1138, Centre de Recherche des Cordeliers, Sorbonne Universite, Universite de Paris, 15-rue de l’ecole de medecine, 75006 Paris, France; 6 Team “Metabolism, Cancer & Immunity”, equipe 11 labellisee par la Ligue contre le Cancer, Paris, France; 7 Department of Molecular Biosciences, The Wenner Gren Institute, Stockholm University, Stockholm, Sweden; 8 BioTechMed Graz, Graz, Austria; 9 Department of Basic Sciences, Faculty of Medicine, University of Crete, 71110 Heraklion, Crete Greece; 10 Pole de Biologie, Hopital Europeen Georges Pompidou, AP-HP, Paris, France; 11 Suzhou Institute for Systems Medicine, Chinese Academy of Sciences, Suzhou, China; 12 Karolinska Institute, Department of Women’s and Children’s Health, Karolinska University Hospital, Stockholm, Sweden

**Keywords:** Cell biology, Biomarkers

## Abstract

Recently, we reported that, in mice, hunger causes the autophagy-dependent release of a protein called “acyl-CoA-binding protein” or “diazepam binding inhibitor” (ACBP/DBI) from cells, resulting in an increase in plasma ACBP concentrations. Administration of extra ACBP is orexigenic and obesogenic, while its neutralization is anorexigenic in mice, suggesting that ACBP is a major stimulator of appetite and lipo-anabolism. Accordingly, obese persons have higher circulating ACBP levels than lean individuals, and anorexia nervosa is associated with subnormal ACBP plasma concentrations. Here, we investigated whether ACBP might play a phylogenetically conserved role in appetite stimulation. We found that extracellular ACBP favors sporulation in *Saccharomyces cerevisiae*, knowing that sporulation is a strategy for yeast to seek new food sources. Moreover, in the nematode *Caenorhabditis elegans*, ACBP increased the ingestion of bacteria as well as the frequency pharyngeal pumping. These observations indicate that ACBP has a phylogenetically ancient role as a ‘hunger factor’ that favors food intake.

## Introduction

Approximately 40% of the adult population of the United States is obese, and other countries follow the same trend or even attain higher proportions^1,2^. Logically, an entire industry proposes strategies for the behavioral, nutritional, and pharmacological treatment of obesity, a condition that nowadays is considered as a disease^3^, even before it leads to metabolic syndrome and entails co-morbidities including diabetes, non-alcoholic fatty liver, atherosclerosis, and cancer^4^. Nowadays, obesity can be considered as the epidemiologically most important risk factor for premature aging, causing a marked reduction in both health span and lifespan^5–9^.

In spite of an ever-expanding scientific literature, current knowledge on the pathogenesis of excessive appetite is limited, and research performed in rodents has not been translated to human obesity. Thus, the first appetite-controlling hormone that has been characterized in mice, leptin^10^, is usually overabundant in obese persons, leading to the proposal of a “leptin resistance” that would account for the obesity-related hyperphagy^11,12^. Only a handful of obese patients bear genetic aberrations in leptin and its receptors that are equivalent to those encountered in *Ob/Ob* and *Db/Db* mice, which lack leptin or its receptor, respectively^13–15^. Similarly, a major appetite-stimulating hormone, ghrelin^16^, is paradoxically low in obese individuals^17,18^.

Recently, we identified acyl-CoA-binding protein (ACBP), also known as diazepam binding inhibitor (DBI), as a novel appetite stimulating factor^19^. Indeed, plasma concentrations of ACBP are elevated in obese patients, as well as in mice that were rendered obese by a high-fat diet or that became obese on a normal diet due to the *Ob*/*Ob* mutation. Neutralization of ACBP by suitable antibodies reduced multiple obesity-related aberrations including increased nutrient uptake, stimulated lipo-catabolism (lipolysis, triglyceride breakdown, fatty acid oxidation, and conversion of glycerol into glucose) and suppressed lipo-anabolism, thus reducing body weight, adiposity, diabetes, and steatosis. These findings could be recapitulated by inducible knockout of the *Dbi* gene. Thus, in contrast to the leptin and ghrelin systems, ACBP appears to play a convergent (rather than divergent) role in the obesity-associated hyperphagy of humans and rodents^19^.

ACBP is a small (13 kDa), phylogenetically conserved protein (Supplemental Fig. 1) that can be found in some eubacteria as well as all three eukaryotic kingdoms (plants, fungi and animals), meaning that it is more ancestral than leptin and ghrelin^20,21^. ACBP has the peculiarity to be secreted as a leaderless protein through a non-conventional (Golgi-dependent) pathway that depends on autophagy^22–24^. In human and mouse cells, ACBP also regulates autophagy. Both the depletion of intracellular ACBP and its addition to the extracellular milieu inhibit autophagy, suggesting that the autophagy-related translocation of ACBP from the intracellular to the extracellular compartment acts as a feedback control system to limit autophagy^19^.

Here, we investigated the possibility that ACBP would act as phylogenetically conserved regulator of autophagy and appetite in two model systems; namely, in the yeast *Saccharomyces cerevisiae* (that undergoes sporulation to seek new food sources) and the nematode *Caenorhabditis elegans* (which can actively search for food and accelerate pharyngeal pumping). We show that ACBP plays an evolutionarily ancient role in appetite control.

## Results

### Opposed autophagy-regulatory effects of ACBP in unicellular and multicellular organisms

**Fig. 1:**
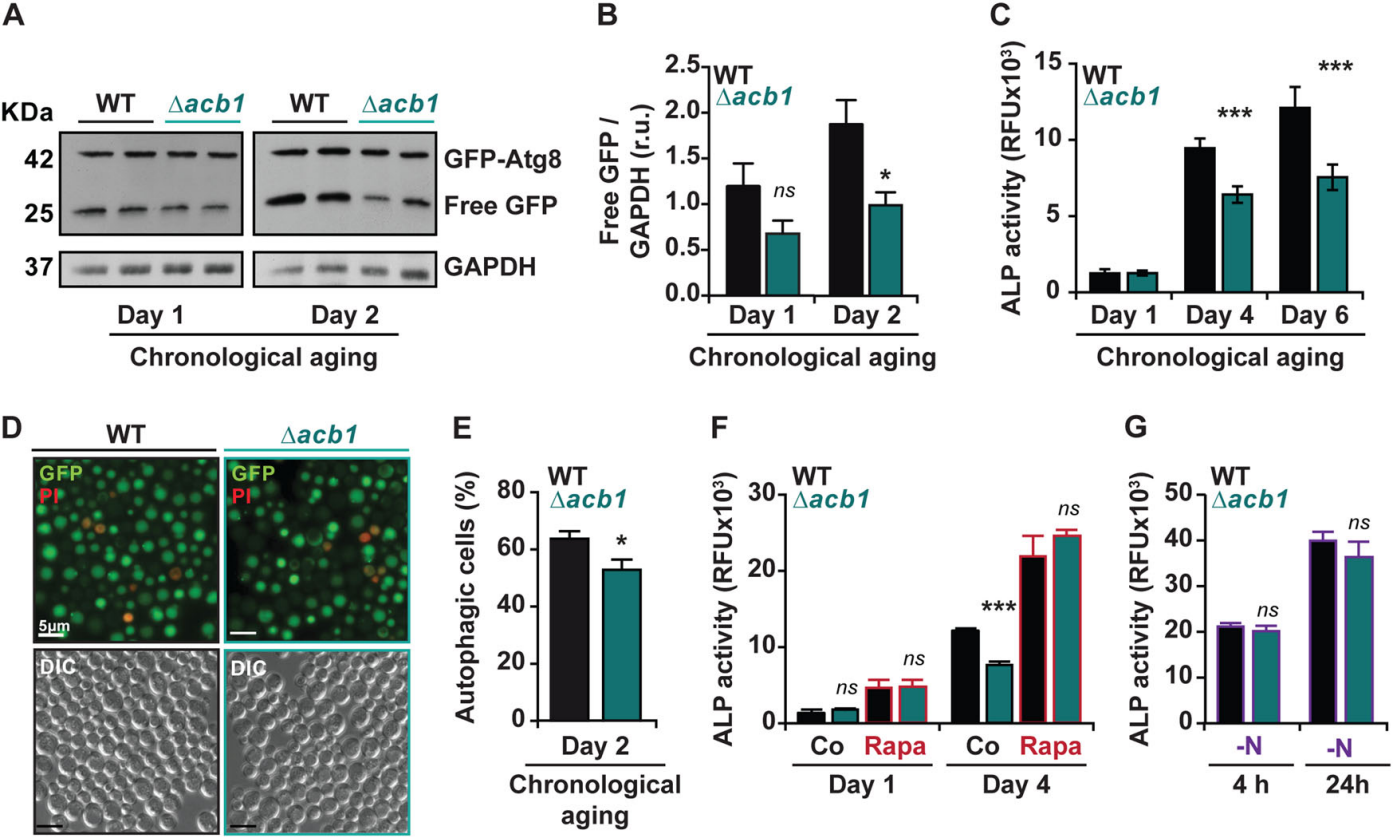
Autophagy regulation by ACBP in *Saccharomyces cerevisiae*. **a**, **b** Immunoblotting analysis of protein extracts from wild type (WT) and *∆acb1* cells expressing a GFP-Atg8 fusion protein. Blots were probed with antibodies against GFP to detect GFP-Atg8 and free GFP, which is indicative of autophagic flux, and against GAPDH as loading control. Representative results (**a**) and densitometric quantification (**b**) at 1 and 2 days are shown. (*n* = 4). **c** Relative alkaline phosphatase (ALP) activity at 1, 4, and 6 days of chronological aging of WT and *∆acb1* cells expressing Pho8pΔN60 (*n* = 3). **d**, **e** Fluorescence microscopy of WT and *∆acb1* cells expressing a GFP-Atg8 chimera at day 2 of chronological aging. Propidium iodide (PI) counterstaining served to visualize dead cells. Scale bar = 5 μm. Autophagic cells were defined as cells with clear vacuolar GFP fluorescence and depicted as percentage of viable (PI^−^) cells. Per strain and replicate, 500–650 cells were manually counted. (*n* = 5). **f**, **g** ALP activity of WT and ∆*acb1* cells expressing Pho8pΔN60 at the indicated times of chronological aging with or without 40 nM rapamycin (Rapa) (**f**) or upon nitrogen starvation (−N) for 4 h and 24 h (**g**) (*n* = 3–5). Quantitative results are reported as means ± SEM. Symbols indicate statistical (Student’s *t*-test) comparisons with controls (n.s, not significant; *p < 0.05, ***p < 0.001).

Knockout of *S. cerevisiae ACB1* (the yeast of ACBP) inhibited autophagy during chronological aging (Fig. 1a–e), although this knockout did not affect maximum autophagy stimulated by rapamycin (Fig. 1f) or nitrogen starvation (Fig. 1g), as determined by assessing the proteolysis of green fluorescent protein (GFP) fused to autophagy-related gene 8 protein (GFP-Atg8) to free GFP detectable by immunoblot (Fig. 1a, b), the enzymatic activity of alkaline phosphatase (ALP) Pho8 (Fig. 1c, f, g), or the redistribution of a GFP-Atg8 to the yeast vacuole detectable by fluorescence microscopy (Fig. 1d, e). Thus, in yeast, Acb1 acts as a facilitator of autophagy.

**Fig. 2:**
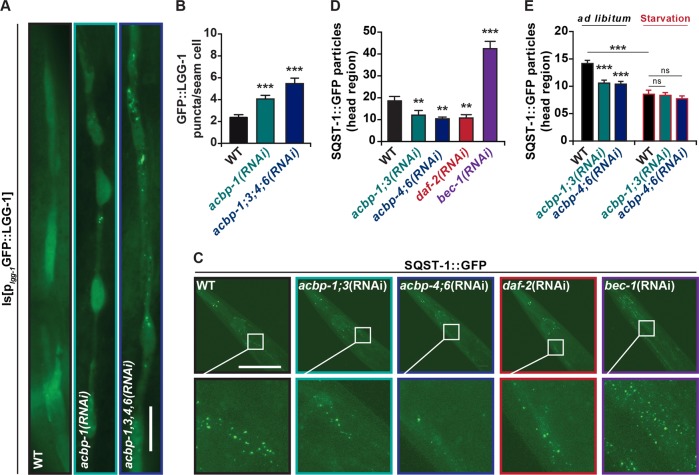
Autophagy regulation by ACBP in *C. elegans*. **a** Representative images of reporter worms expressing a GFP::LGG-1 fusion protein in the WT and genetic backgrounds lacking indicated *acbp* family genes (single *acbp-1(sv62)* and quadruple *acbp-1*(*sv62*);*acbp-3*(*sv73*)*;acbp-4*(*tm2896*)*;acbp-6*(*tm2995*) mutants. Scale bar, 20 μm. **b** Quantification of GFP::LGG-1 puncta per hypodermal seam cell in WT, *acbp-1(sv62)* and *acbp-1*(*sv62*);*acbp-3*(*sv73*)*;acbp-4*(*tm2896*)*;acbp-6*(*tm2995*) mutant animals (*n* = 25 worms). **c** Representative images of SQST-1::GFP reporter worms treated with known regulators of autophagy (*daf-2* and *bec-1*), as well as indicated *acbp* RNAis. Scale bar, 100 μm. **d** Quantification of SQST-1/p62::GFP puncta in the head region in control and RNAis treated animals (*n* = 25 worms). **e** Quantification of SQST-1/p62::GFP puncta in the head region in control and RNAi treated animals fed ad libitum or upon 12 h of starvation (*n* = 25 worms). Quantitative results are reported as means ± SEM. Symbols indicate statistical (Student’s *t*-test) comparisons with controls (n.s, not significant; **p < 0.01, ***p < 0.001). All experiments were repeated at least three times, yielding similar results.

In sharp contrast, knockout of *C. elegans acbp-1* (the nematode orthologous of ACBP), alone or together with several homologs *acbp-3, acbp-4* and/or *acbp-6* (which exist in this species but not in yeast nor in mammals)^25^, stimulated autophagy, as indicated by the subcellular redistribution of a GFP::LGG-1 fusion protein (LGG-1 is the nematode orthologous of yeast Atg8 and mammalian LC3) to cytoplasmic puncta (Fig. 2a, b) and the concomitant decrease of SQST-1/p62::GFP (the nematode orthologous of mammalian SQSTM1 fused to GFP) puncta (Fig. 2c, d). Knockdown of *daf-2* (the insulin/insulin growth factor 1 receptor) which induces autophagy^26^ also decreased SQST-1/p62::GFP puncta, while knockdown of *bec-1* (the nematode orthologous of mammalian BECN1) robustly increased them, proving that this reporter can be reliably utilized for measuring autophagic flux (Fig. 2c, d). Twelve hours of starvation led to a similar decrease of SQST-1::GFP particles in control animals and *acbp-1;3* and *acbp-4;6* knockout worms (Fig. 2e). Of note the increase in autophagy induced by deletion of *acbp* genes was partially reduced by mutation of *aak-2* (an orthologous of human PRKAA1 and PRKAA2, which encode subunits of AMP activated kinase, AMPK) which is implicated in autophagy induction via ULK-1 phosphorylation^27^. However, knockdown of *acbp* genes was unable to induce a further increase in autophagy in *daf-2* mutants (which lack a functional insulin/insulin growth factor 1 receptor) (Supplemental Fig. 2). Thus, in nematodes, *acbp* genes act as endogenous inhibitors of autophagy.

This discrepancy between yeast and worms suggests that ACBP might have distinct autophagy-regulatory functions in unicellular vs. multicellular systems.

### Convergent effects of ACBP depletion on feeding behavior in yeast and nematodes

In the next step, we determined whether the effect of ACBP on feeding behavior is phylogenetically conserved.

**Fig. 3:**
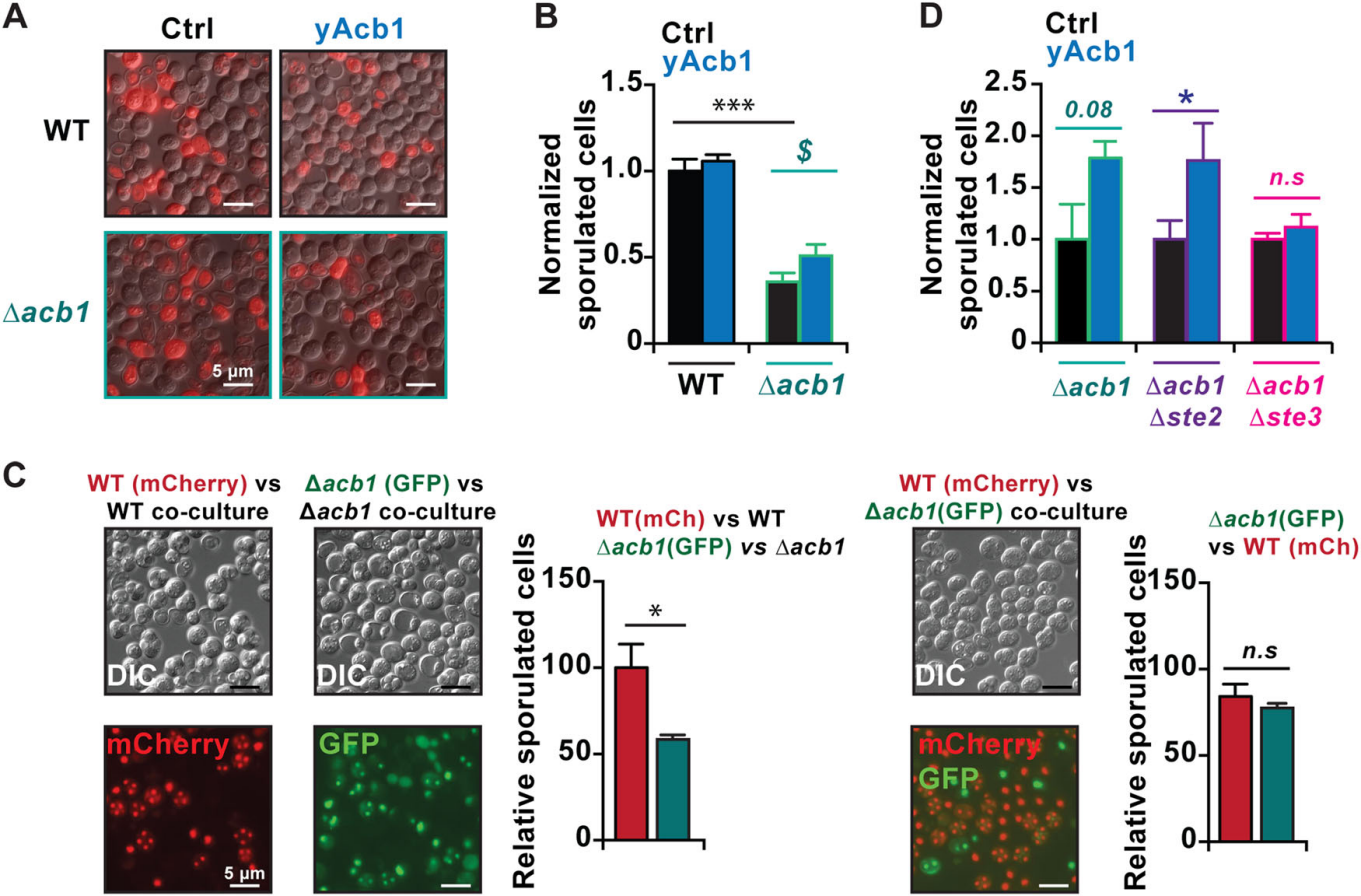
Role of Acb1 in the sporulation of *S. cerevisiae*. **a**, **b** Representative pictures and quantification of sporulation frequencies of wild type (WT) and Δ*acb1* cells with or without the addition of recombinant yeast Acb1 (yAcb1). Addition of yAcb1 to Δ*acb1* cultures partially reversed the sporulation frequency defect induced by the *ACB1* deletion. **c** Representative pictures and quantification of sporulation frequencies of yeast cells with the indicated genotypes that were co-cultured are shown. Note that co-culturing of the Δ*acb1* strain (Hho1-GFP tagged) with a WT strain (Hho1-mCherry tagged) reduced the sporulation defect of this mutant. **d** Additional deletion of *STE3* (coding for membrane receptor, that couples factor a pheromone binding to a MAP kinase cascade) renders mutants resistant to external yAcb1 regarding sporulation frequency, whereas deletion of *STE2* (coding for alpha factor membrane receptor) did not affect the response to extracellular yAcb1. Results are expressed as means ± SEM (*n* = 3–6). n.s, not significant; *p < 0.05, ***p < 0.0001 (Student’s *t*-test), as compared to untreated controls (Ctrl) or ^**$**^*p* < 0.05 (Student’s *t*-test), as compared within *acb1* deficient conditions.

Yeast is devoid of ameboid movement, and the only possibility for this organism to seek new sources of nutrients consists in sporulation, which occurs in response to prolonged exhaustion of external resources^28^. The knockout of *S. cerevisiae ACB1* (∆*acb1*) led to a defect in sporulation and this sporulation defect of ∆*acb1* cells was blunted by adding recombinant yeast Acb1 (yAcb1) protein to the cultures (Fig. 3a, b), confirming that Acb1 stimulates sporulation. To investigate possible non-cell-autonomous effects of ACBP on sporulation, we co-cultured WT Hho1-mCherry tagged cells (which emits a red fluorescence) with ∆acb1 Hho1-GFP cells (expressing green fluorescent protein (GFP)). As controls these fluorescent protein tagged strains were co-cultured with respective non-fluorescent variants. This procedure revealed that ∆*acb1* yeast co-cultured with ∆*acb1* yeast cells exhibit a sporulation defect that is attenuated in the presence of WT cells (Fig. 3c). Thus, the sporulation defect of ∆*acb1* cells could not only be partially rescued by adding recombinant yAcb1 protein to the cultures but was also blunted by co-culturing the cells with Acb1-expressing yeast cells (Fig. 3a–c), confirming that Acb1 stimulates sporulation. Finally, the rescue of the sporulation defect by Acb1 protein depended on Ste3 (but not Ste2), which is one of the two G protein-coupled receptors encoded by the yeast genome (Fig. 3d). These results indicate that extracellular Acb1 protein can act on Ste3 receptors to stimulate sporulation in yeast.

**Fig. 4:**
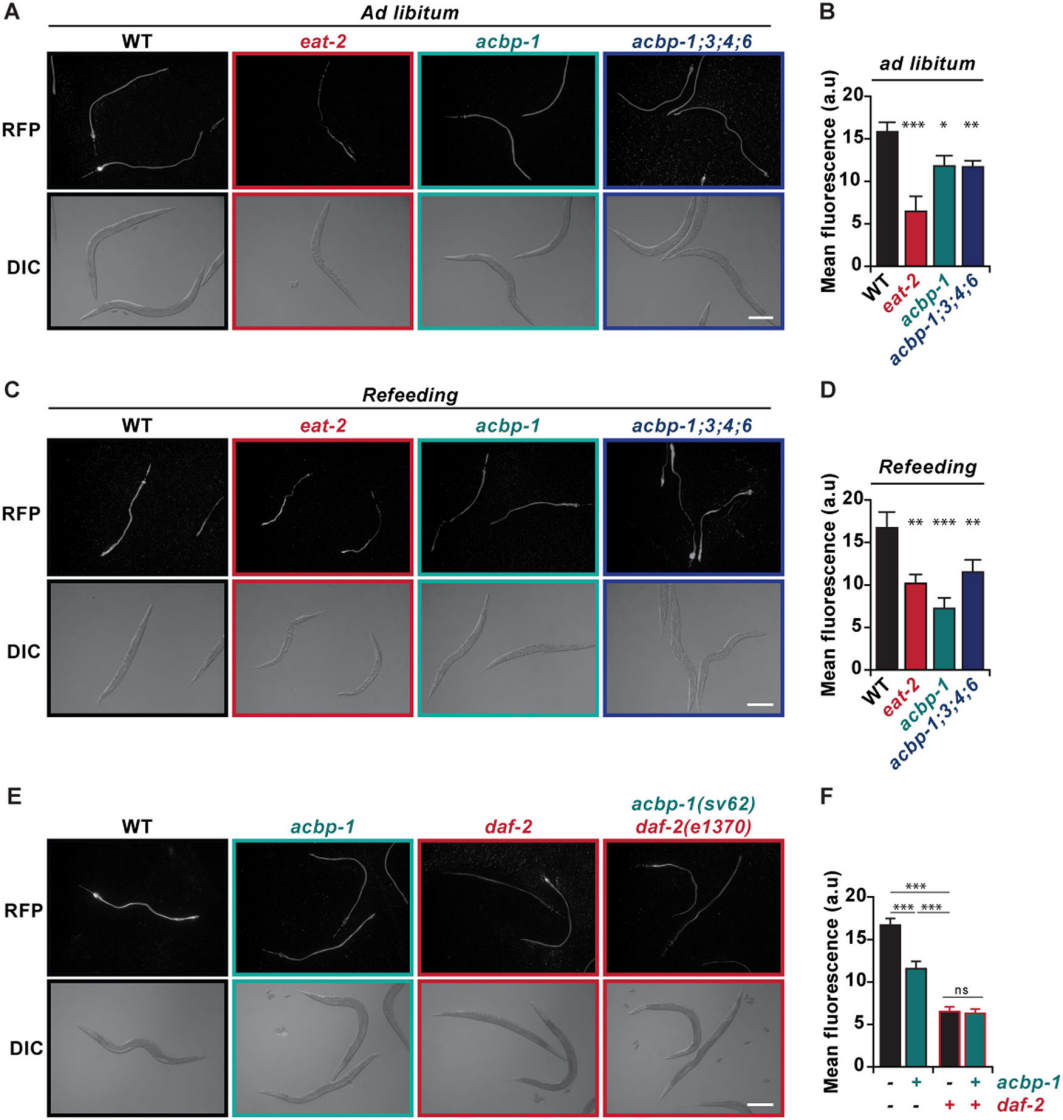
ACBP family members regulate food intake in *C. elegans*. Fluorescent images (**a**, **c**, **e**) and quantification of intestinal fluorescence intensity (**b**, **d**, **f**) of D1 adult animals of the respective genotypes (*n* = 20 per genotype) fed with RFP fluorescent bacteria for 5 min. ACBP deficient animals exhibit a decrease in food intake compared to their WT counterparts when fed *ad libitum* (**a**, **b**, **e**, **f**) and after 12 h of starvation (**c**, **d**). *eat-2(ad465)* mutants were used as a positive control for reduced food uptake. All experiments were repeated at least three times, yielding similar results. Quantitative results are reported as means ± SEM. Symbols indicate statistical (Student’s *t*-test) comparisons (n.s, not significant; *p < 0.05, **p < 0.01, ***p < 0.001), as compared to WT animals (**b**, **d**) or between the indicated conditions (**f**). Scale bar, 200 μm.

Next, we turned to the nematode model. In *C. elegans*, knockout of one or several genes coding for ACBP orthologous (*acbp-1, acbp-3, acbp-4*, and/or *acbp-6*) reduced the uptake of bacteria expressing red fluorescent protein (RFP) both in ad libitum feeding conditions (Fig. 4a, b) and after 12 h of starvation and refeeding (Fig. 4c, d). This result was confirmed by the analysis of pharyngeal pumping, revealing that removal of *acbp-1, acbp-3, acbp-4*, and/or *acbp-6* reduced food intake (Supplemental Fig. 3) in *C. elegans*. The generation of *acbp-1(sv62)*;*daf-2(e1370)* double mutants revealed that the *daf-2* mutation is epistatic to the *acbp-1* mutation, since the *acbp-1;daf-2* double mutants behave quite similarly to single *daf-2* mutants in respect to feeding (Fig. 4e, f). These observations indicate that the worm orthologous of ACBP stimulate feeding behavior, as additionally corroborated by comparisons with *tax-4(p678)* mutants (Fig. S4A, B) or animals treated with clozapine (Fig. S4C, D). TAX-4 is a nucleotide-gated channel which is broadly expressed in the *C. elegans* nervous system and in ASI neurons which mediate satiety quiescence^29,30^, while clozapine is a second generation antipsychotic drug, which inhibits pharyngeal pumping of nematodes^31^. Of note, *acbp-1(sv62)* mutants seem more sensitive to clozapine administration compared to their wild-type counterparts (Fig. S4C, D).

## Discussion

ACBP is an evolutionarily ancient protein, based on sequence alignments and structural similarities suggesting that the physicochemical properties of this protein have been conserved throughout the eukaryotic radiation^20,21,32^. The present data suggest that the function of ACBP as a regulator of appetite is phylogenetically conserved as well.

At a first level, knockout of the gene coding for the (single) ACBP orthologous from yeast reduces sporulation, while its addition in the form of a recombinant protein restores sporulation, presumably through an action on Ste3, which is one of the two signal-transducing receptors present in this species. Ste3, a seven transmembrane G protein-coupled receptor, is best known to for the peptide pheromone alpha1-factor^33^, the mating factor of yeast, pointing to an intriguing cross-talk between the signal transduction pathways involved in mating and in the control of food intake. At a second level, knockout of the genes coding for the (several) ACBP orthologous from *C. elegans* reduces the uptake of bacteria as it reduces the frequency of pharyngeal pumping. Together, these findings favor the contention that the appetite control function of ACBP is conserved throughout evolution. Indeed, these results echo a previous report on a gene named *Anorexia* (*Anox*) that codes for an acyl-CoA-binding protein with an ankyrin repeat domain and that, if mutated, reduces feeding activity and mouth hook movement (the fly equivalent of mastication) in *Drosophila melanogaster*^34^. That said, *Anox* has been involved in central appetite control because it is mostly expressed in the central nervous system and in ganglions, differing from mouse ACBP that has been attributed a predominantly peripheral role in appetite control^19^.

The effects of ACBP on autophagy appear to be distinct in yeast and in nematodes. Thus, removal of the ACBP orthologous from yeast inhibited autophagy during chronological aging, contrasting with the observation that, in *C. elegans*, deletion of ACBP orthologous resulted in enhanced autophagy. As a possibility, these seemingly contradictory results reflect the distinct cellular organization of these species (monocellular for yeast, multicellular for nematodes), as well as the differential effects of intracellular vs. extracellular ACBP on autophagy. Indeed, when (mostly intracellular) ACBP is depleted from cultured human cells by RNA interference, this results in autophagy inhibition. However, when (mostly extracellular) ACBP is neutralized by antibodies, this results in autophagy induction, both in cultured human cells and in mice^19^. Thus, removal of ACBP from *S. cerevisiae* might reflect a situation in which physiological effects are secondary to the depletion of intracellular ACBP, while removal of ACBP from *C. elegans* might reflect the effects of a reduction in extracellular ACBP.

Irrespective of the aforementioned uncertainties, it appears clear that ACBP plays a major appetite-stimulatory role throughout eukaryotic evolution, meaning that it triggers a range of different feeding behaviors in yeast (sporulation), nematodes (pharyngeal pumping), insects (mouth hook movement) and mammals (food intake). Since it operates independently from the leptin and ghrelin systems (which only exist in mammals)^35,36^, ACBP may indeed represent the phylogenetically most ancient “hunger factor”. In a plausible scenario, starvation causes autophagy, resulting in the release of ACBP from cells, and ACBP then acts on cell surface receptors to stimulate feeding behaviors. In this sense, ACBP would act as a neuroendocrine factor that participates in a primate homeostatic feedback loop or ‘hunger reflex’ designed to mitigate the effects of nutrient deprivation.

## Materials and methods

### *C. elegans* experiments

We followed standard procedures for maintaining *C. elegans* strains. The rearing temperature was set at 20 °C for all experiments. We used the DA2123: adIs2122 [p_*lgg-1*_GFP::LGG-1+rol-6(su1006)] to measure LGG-1/LC-3 autophagic puncta and the HZ589: *him-5(e1490)*V;bpIs151 [p_*sqst-1*_SQST-1::GFP+unc-76(+)] to measure SQST-1/p62 puncta as previously described^27,37^. The DA2123 strain was crossed with the SV62:*acbp-1(sv62)*I and the quadruple FE0017:*acbp-1(sv62)*I;*acbp-6(tm2995)*II;*acbp-4(tm2896)*III;*acbp-3(sv73)*X strains to monitor autophagy in the *acbp* family mutant genetic backgrounds^25^. For pharyngeal pumping measurements, the SV62 and FE0017 strains were compared with DA465: *eat-2(ad465)*II, a genetic model for reduced pharyngeal pumping, PR678: *tax-4(p678)*III and CB1370: *daf-2(e1370)*III mutants. The following strains were generated in the present study:

IR1780: *acbp-1(sv62)*I; adIs2122 [p_*lgg-1*_GFP::LGG-1+rol-6(su1006)]

IR1792: *acbp-1(sv62)*I;*acbp-6(tm2995)*II;*acbp-4(tm2896)*III;*acbp-3(sv73)*X; adIs2122 [p_*lgg-1*_GFP::LGG-1+rol-6(su1006)]

IR2686: *acbp-1(sv62)*I; *daf-2(e1370)*III.

Autophagy was measured as described in the literature^38^. For measuring LGG-1/LC-3 puncta, 10 well-fed adult worms of the respective genetic backgrounds were allowed to lay eggs on nematode growth medium or RNAi plates. Four hours later, parents were removed and plates were placed at 20 °C. 2.5 days later, synchronized animals were collected, anaesthetized with 10 mM levamisole and mounted on slides for microscopic examination. The number of GFP::LGG-1 positive autophagic puncta was counted in hypodermal seam cells at the late L3–L4 larval stages^26^.

Pharyngeal pumping was measured as previously described^39^. Grinder movements of free-moving animals were measured under the stereomicroscope. Three independent measurements were performed for each individual and the average number of pumps per animal was recorded. Starvation was performed by placing the animals on NGM plates without bacterial lawn for 12 h. For assessing food intake using fluorescent bacteria, we fed synchronized day one (D1) adult animals for 5 min with HT115 bacteria transformed with a IPTG-inducible RFP expressing plasmid (modification of a previous protocol published^40^). Upon this short feeding period, the animals were immediately immobilized with levamisole and mounted on slides for microscopic observation with a Zeiss AxioImager Z2 epifluorescence microscope. Image J software was used for the quantification of mean RFP intestinal fluorescence. Clozapine (Sigma-Aldrich, product number: C6305) was diluted in 100% ethanol (stock 11 mg/mL) and was added before pouring of NGM plates at a final concentration of 200 μg/mL per plate, as previously described^31^. Plates with solvent alone (1.8% ethanol per plate) were used for comparison. Synchronized animals at day 1 of adulthood were placed overnight (14–16 h) on clozapine or ethanol-containing plates to avoid undesired developmental effects which have been previously described^31^). Ingestion of RFP+ bacteria at day 2 adult animals was measured as described above.

The following sets of primers were used both for the construction of *acbp* RNAi constructs and the detection of *acbp* gene deletions:

*acbp-1* FW: 5′-TTGCAGAATTTTGCGAGTTTC-3′

*acbp-1* REV: 5′-AGAATTTATTTAGGCTCCGTACTTG-3′

*acbp-3* FW: 5′-TTAGGTCAACAGCAGCAGCC-3′

*acbp-3* REV: 5′-ACACACATAACTCACGCAATTCTGA-3′

*acbp-4* FW: 5′-CGATTATTCTGTTTTAGAGTGTTTGA-3′

*acbp-4* REV: 5′-GAAGTGCTCACGGAGTTGATT-3′

*acbp-6* FW: 5′-ACGCCCCATAATAGTAAAAGATGC-3′

*acbp-6* REV: 5′-AAACATTCCCCATTTCTCTATCTCTC-3′.

The respective genomic fragments were initially cloned in TOPO and then in pL4440 vector backbone in combinations to generate double *acbp* RNAi constructs (*acbp-1* together with *acbp-3* and *acbp-4* with *acbp-6*).

### *S. cerevisiae* experiments

#### Strains

Wild type *S. cerevisiae* strain L5366 (wild type, *MAT*a/α *ura3-52/ura3-52* in the Σ1278b strain background^41^,.pngt from Dr. A. H. Limper) and the double deletion strain L5366 Δ*acb1* (Δ*acb1*::*URA3*/Δ*acb1*::*URA3*) as well as Hho1 GFP-tagged or mCherry-tagged variants thereof (L5366 mCherry and L5366 Δ*acb1* GFP) were used. *HHO1* codes for the Histone H1 protein. Analyses of autophagy in yeast were carried out in BY4742 (MATα *his3Δ1 leu2Δ0 lys2Δ0 ura3Δ0*) and respective ∆*acb1* mutant obtained from EUROSCARF. Strains were grown at 28 °C on synthetic minimal medium containing 0.17% yeast nitrogen base (Difco), 0.5% (NH_4_)_2_SO_4_ and 30 mg/L of all amino acids (except 80 mg/L histidine, 120 mg/L lysine, and 200 mg/L leucine), 30 mg/L adenine and 320 mg/L uracil with 2% glucose. For chronological aging, cells were inoculated to OD_600nm_ 0.1 from fresh overnight cultures and grown at 28 °C. Where indicated, cultures were supplemented with 40 nM rapamycin (AG Scientific; 1 mg/mL stock in DMSO) at the time point of inoculation. For nitrogen starvation, cells where grown to OD_600nm_ 1 on synthetic minimal medium and then transferred to medium without amino acids and (NH_4_)_2_SO_4_.

#### Yeast strain construction

Deletions of *ACB1* were made in the haploid variants after sporulation of L5366 following standard protocols with PCR generated cassettes using primers ACB1_fw (5′-GAAGACTAAAACTCTAAAATTAGTTAAACTAGTGTTTTCAGCAAAcagctgaagcttcgtacgc -3′) and ACB1_rev (5′-AAAGCTAGGCCAAAACTCCTTACATGGAGCTAGTATACCCCTTTTgcataggccactagtggatc-3′) and pUG72 (URA3 marker) as template^42,43^ Transformation was done using the lithium acetate method^44^. Deletion was verified by PCR (Primer ACB1_ctrl 5′-GTCTAGCAATTTGTGTAGGGACT and plasmid-corresponding control Primer^42,45^. The generated haploid knock-out strains were transformed with a mating vector for selection purposes—*Mat*a with pIS419 and *Mat*α with pIS420 (EUROSCARF^46^)—and mated. Selection of diploid strains was performed by plating on agar plates containing both clonNAT (100 µg/mL, Sigma = wrong Werner BioAgents) and Hygromycin (300 µg/mL, Sigma = wrong InvivoGen). Diploid strains were grown and transferred several times on YPD-medium to encourage plasmid loss and plated afterwards on YPD agar plates. Occurring colonies were tested on agar plates containing Hygromycin, clonNAT or both Hygromycin and clonNAT for loss of the respective plasmid. Deletion of *STE2* or *STE3* in this diploid *acb1* deletion strain was done by linear transformation with PCR-generated cassettes using pFA6a-hphNT1 (Hygromycin) and pFA6a-natNT2 (clonNAT) as templates as described above (Primers: STE2_S1_fw:GTTACTTAAAAATGCACCGTTAAGAACCATATCCAAGAATCAAAAATGCGTACGCTGCAGGTCGAC; STE2_S2_rev: TCAAAATTTACGGCTTTGAAAAAGTAATTTCGTGACCTTCGGTATTTAA TCGATGAATTCGAGCTCG; STE2_ctrl: AGTGCTCGAATAGGTGTTGC; STE3_S1_fw: AGGCAATTAAAT TTGTGTAGGAAAGGCAAAATACTATCAAAATTTTCATGCGTACGCTGCAGGTCGAC; STE3_S2_rev: AA AATAAAATACTCCTAGTCCAGTAAATATAATGCGACACTCTTGTGTTAATCGATGAATTCGAGCTCG; STE3_ctrl: GTACCACATTGCCAGATTTATGA).

To follow sporulated cells during co-culturing, fluorescent tags (mCherry and GFP, respectively) were recombinated to the histone H1 gene (*HHO1*) in the haploid WT L5366 and Δ*acb1*::*URA3* strains following standard protocols with PCR generated cassettes using primers Hho1_S3_fw (5′-AAGGGCCCCTCCGGCATTATTAAACTAAACAAGAAGAAGGTCAAACTCTCCACGCGTACGCTGC-3′) and Hho1_S2_rv (5′-TTTGATAGTATTGCTATCACCATTGACATTCTCGTTTGGATATTCACTTTTTAATCGATGAATTCG-3′) and templates pFA6a 3mCherry-hphNT1 and pFA6a 3mCherry-natNT2 (for mCherry tag) or pYM12 and pYM25 (for GFP-Tag). Deletion was verified by PCR (Primer Hho1_ctrl 5′-AGCATGCCTCAACTTAATGAC-3′ and plasmid-corresponding control Primer^47^). The haploid strains were mated afterwards and selected on agar plates containing Hygromycin and Geneticin or ClonNat, respectively.

#### Autophagy measurement in yeast

Autophagy was analyzed using two different approaches: Alkaline phosphatase (ALP) activity was determined as previously described^47^. Briefly, cells were transformed with and selected for stable insertion of a pTN9 *Hind*III fragment containing an engineered construct of *PHO8* which codes for a protein that lacks its N-terminal transmembrane domain (Pho8pΔN60). ALP activity was measured at indicated days of chronological aging using 1 µg of total protein as determined via BioRad protein assay (BioRad). In order to correct for intrinsic (background) ALP activity, respective strains without pTN9 insertion were simultaneously processed and obtained values were subtracted as background. In addition, autophagy was monitored using cells equipped with a pUG36-URA plasmid coding for a GFP-Atg8 fusion protein as previously described^48^. At day 2 of chronological aging, cells were counterstained with 0.1 µg/mL propidium iodide (PI) to exclude dead cells and analyzed for GFP-Atg8 localization via fluorescence microscopy using small-band eGFP and DsRed filters (Zeiss) on a Zeiss Axioskop microscope. To monitor GFP liberation indicative of autophagic vacuolar breakdown of GFP-Atg8, cells were subjected to chemical lysis followed by SDS-PAGE and western blot using standard protocols^49^. Blots were probed with anti-GFP (Roche, #11814460001) and anti-glyceraldehyde 3-phosphat dehydrogenase (GAPDH;.pngt from Sepp Kohlwein, University of Graz) antibodies and the respective peroxidase-conjugated secondary antibodies (Sigma). Densitometric quantification was performed with Image Lab 5.2 Software (Bio-Rad). Quantification and statistical analysis: Micrographs of cells expressing GFP-Atg8 were manually counted, and 500–650 cells were evaluated per strain and per experiment. Thereby, cells displaying clear vacuolar GFP fluorescence were scored as autophagic cells and were depicted as percentage of viable (PI negative) cells. Data represents mean of five independent experiments. Densitometric quantification of immunoblots was performed with Image Lab 5.2 Software (Bio-Rad), and the ratio Free GFP/GAPDH was plotted. Data represent mean ± SEM of four independent experiments. Data showing ALP activity represent mean ± SEM of three independent experiments. Statistical analyses were performed using Students *T*-test (one-tailed, unpaired), with *p < 0.05, **p < 0.01, and ***p < 0.001.

#### Sporulation assay

For sporulation experiments all strains (L5366 and acb1 deletion variant) were grown 21–22 h over night in 2 mL YPD medium containing 1% yeast extract, 2% peptone and 2% glucose at 28 °C. Afterwards, the strains were inoculated to an OD_600_ of 0.3 in 2 mL pre-sporulation YPA medium containing 1% yeast extract, 2% peptone and 3% Potassium acetate^50^ and +/− 2 µg/mL final concentration of yAcb1 (recombinant *S. cerevisiae* Acyl-CoA-binding protein (acb1) from Mybiosource) at 28°. After 18–22 h cells were washed twice with 500 µL ddH_2_O and shifted to an OD_600_ of 0.5 in 2 mL high carbon (HC) sporulation medium (0.2% raffinose, 1% potassium acetate) and +/− 2 µg yAcb1/mL and grown at 25 °C for 3 days.

For sporulation co-culturing experiments with Hho1-fluorescent-tagged strains, cultures were grown 21–22 h over night in YPD medium at 28 °C, like above. Then strains were mixed to an OD_600_ of 0.15 each, in pre-sporulation medium YPA at 28 °C. Hho1-GFP-tagged L5366 Δacb1::URA3/Δacb1::URA3 strains were mixed with Hho1-mCherry-tagged WT-strains and L5366 Δacb1::URA3/Δacb1::URA3 strain, respectively. Correspondingly Hho1-mCherry-tagged WT-strain was also mixed with un-tagged WT-strains. After 18–22 h cells were washed twice with 500 µL ddH_2_O, shifted to an OD_600_ of 0.5 into 2 mL HC sporulation medium and grown at 25 °C for 3 days.

#### Microscopy studies

Microscopy was performed as previously described^51^ In short, for sporulation experiments with addition of yAcb1, cells were harvested after 3 days and stained with PI (0.1 µg/mL in 1×PBS).

Cells were viewed and documented by fluorescence microscopy with the use of a small-band dsRed filter (Zeiss) on a Zeiss Axioskop microscope. Sample images were taken with a Diagnostic Instruments camera (Model: SPOT 9.0 Monochrome-6), acquired and processed (coloring) using the Metamorph software (version 6.2r4, Universal Imaging Corp.) Subsequently, pictures were quantified by evaluating the amount of cells, which were sporulated, and the cells that were stained red (dead cells), relative to all pictured cells. At least 250–900 cells per strain per independent experiment were manually counted.

For sporulation co-culturing experiments with Hho1-tagged mCherry and GFP strains, cells were harvested after 3 days. Cells were viewed and documented by fluorescence microscopy with the use of a small-band eGFP and dsRed filter (Zeiss) on a Zeiss Axioskop microscope. Microscopy pictures were quantified by evaluating the amount of sporulated and un-sporulated cells of wild-type vs. *acb1* deletion strains, compared to their respective mono-cultures. 250–900 cells per strain per experiment were manually counted. Data represent results of four independent experiments. Statistical analyses for sporulation experiments were performed using Students *t*-test (one-tailed, unpaired), with *p < 0.05, **p < 0.01, and ***p < 0.001.

#### Statistical analysis

Data are reported as the mean ± standard deviation (SD), mean ± standard error of the mean (SEM), or Box and whisker plots (mean, first and third quartiles, and maximum and minimum values) as specified. The number of independent data points (*n*) is indicated in the figure legends of the corresponding graphs or in the legends. For statistical analyses, *p* values were calculated by two-way ANOVA, one-way ANOVA with Tukey’s multiple comparisons test, two-tailed unpaired Student’s *t*-test, Wilcoxon matched pairs signed rank test, Pearson’s coefficients of correlation (R) or false discovery rate (FDR) as indicated (Prism version 7, GraphPad Software). Differences were considered statistically significant when *p*-values *(*p* < 0.05), **(*p* < 0.01), ***(*p* < 0.001) and n.s. = not significant (*p* > 0.1).

## Supplementary information

Supplemental Figure Legends

Supplemental Figure S1

Supplemental Figure S2

Supplemental Figure S3

Supplemental Figure S4
