# Supplementary material for: Acyl-CoA-binding protein (ACBP): a phylogenetically conserved appetite stimulator
Source: Cell Death Dis. 2020 Jan 6;11(1):7. doi: 10.1038/s41419-019-2205-x (PMC6944704; doi:10.1038/s41419-019-2205-x)
Supplement: Supplementary file 1 — Supplemental Figure Legends [file 41419_2019_2205_MOESM1_ESM.docx]

**Legends to supplemental figures**

**Supplemental Figure S1. Sequence similarity between ACBP homologous.**

Related proteins were found in *Homo sapiens* (P07108), *Mus musculus* (P31786), *Caenorhabditis elegans* (O01805) and *Saccharomyces cerevisiae* (P31787). Stars represent amino acids that are identical among all 4 homologs, and the points represent conservative or semi-conservative mutations.

**Supplemental Figure S2. ACBP inhibition induces autophagy in a partially AMPK dependent manner.**

(**A**) The number of LGG-1/LC3 positive puncta was measured in wild-type (WT), *aak-2*/AMPK and *daf-2*/IGFR mutant animals upon knockdown of *acbp* genes. ACBP depletion markedly increases autophagy in the WT background and partially in the *aak-2* mutant background but fails to do so in *daf-2* mutants, which display constitutively increased autophagy levels (n=25 animals per condition, ****p*<0.001 compared to the respective control; one-way analysis of variance ANOVA). All experiments were repeated 3 times, yielding similar results.

(**B**) Representative images of hypodermal seam cells in L4 larvae of wild type, *aak-2* and *daf-2* mutant background fed with control bacteria or bacteria expressing RNAi against *acbp* genes. Scale bar, 20 μm.

**Supplemental Figure S3. ACBP family members regulate pharyngeal pumping in *C. elegans*.**

(**A**) DIC image of the *C. elegans* pharynx. The arrowhead highlights the grinder. Scale bar, 20 μm.

(**B**) Quantification of pharyngeal pumping in WT nematodes or mutants lacking *acbp-1* alone, *acbp-1*;*3;4;6* or *eat-2*.

****p*<0.001 (Student’s *t* test), as compared to WT animals. All experiments were repeated 3 times with n=20 animals per condition, yielding similar results.

**Supplemental Figure S4. ACBP family members regulate appetite in *C. elegans* similarly to positive controls for reduced food intake.**

(**A**-**B**) Representative photomicrographs (**A**) and quantification of intestinal fluorescence intensity (**B**) of D1 adult wild-type (WT), *acbp-1*(sv62) and *tax-4*(p678) mutants fed for five minutes with RFP^+^ fluorescent bacteria.

(**C**-**D**) Representative photomicrographs (**C**) and quantification of intestinal fluorescence intensity (**D**) of D2 adult WT and *acbp-1*(sv62) fed for five minutes with RFP^+^ fluorescent bacteria. The animals were grown on plates with 1.8% ethanol (EtOH) or 200μg/mL clozapine.

All experiments were repeated 3 times, yielding similar results. Quantitative results are reported as means ± SEM. Symbols indicate statistical (Student’s *t* test) comparisons (n.s, not significant, **p*<0.05, ***p*<0.01, ****p*<0.001), as compared between the indicated conditions. Scale bar, 200μm.
