# Supplementary figures and images for: Acyl-CoA-binding protein (ACBP): a phylogenetically conserved appetite stimulator
Source: Cell Death Dis. 2020 Jan 6;11(1):7. doi: 10.1038/s41419-019-2205-x (PMC6944704; doi:10.1038/s41419-019-2205-x)

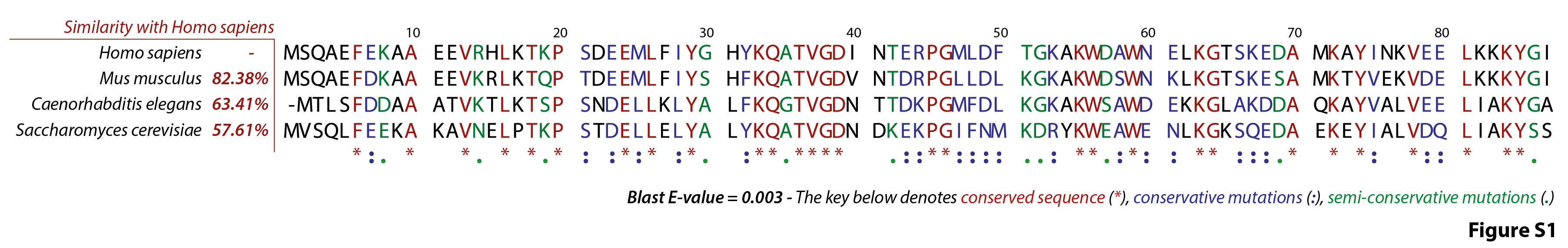

Supplement: Supplementary file 2 — Supplemental Figure S1 [file 41419_2019_2205_MOESM2_ESM.tif]

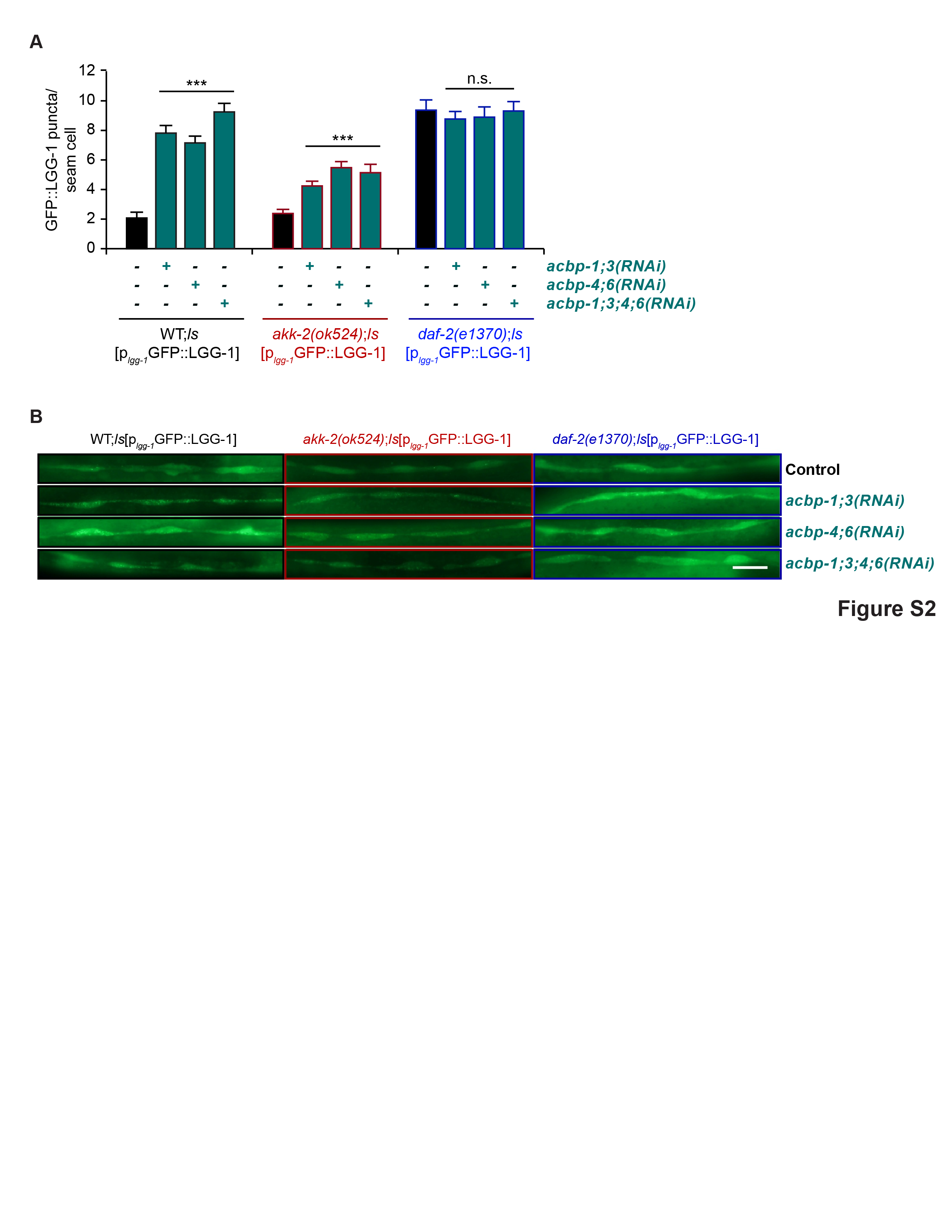

Supplement: Supplementary file 3 — Supplemental Figure S2 [file 41419_2019_2205_MOESM3_ESM.tif]

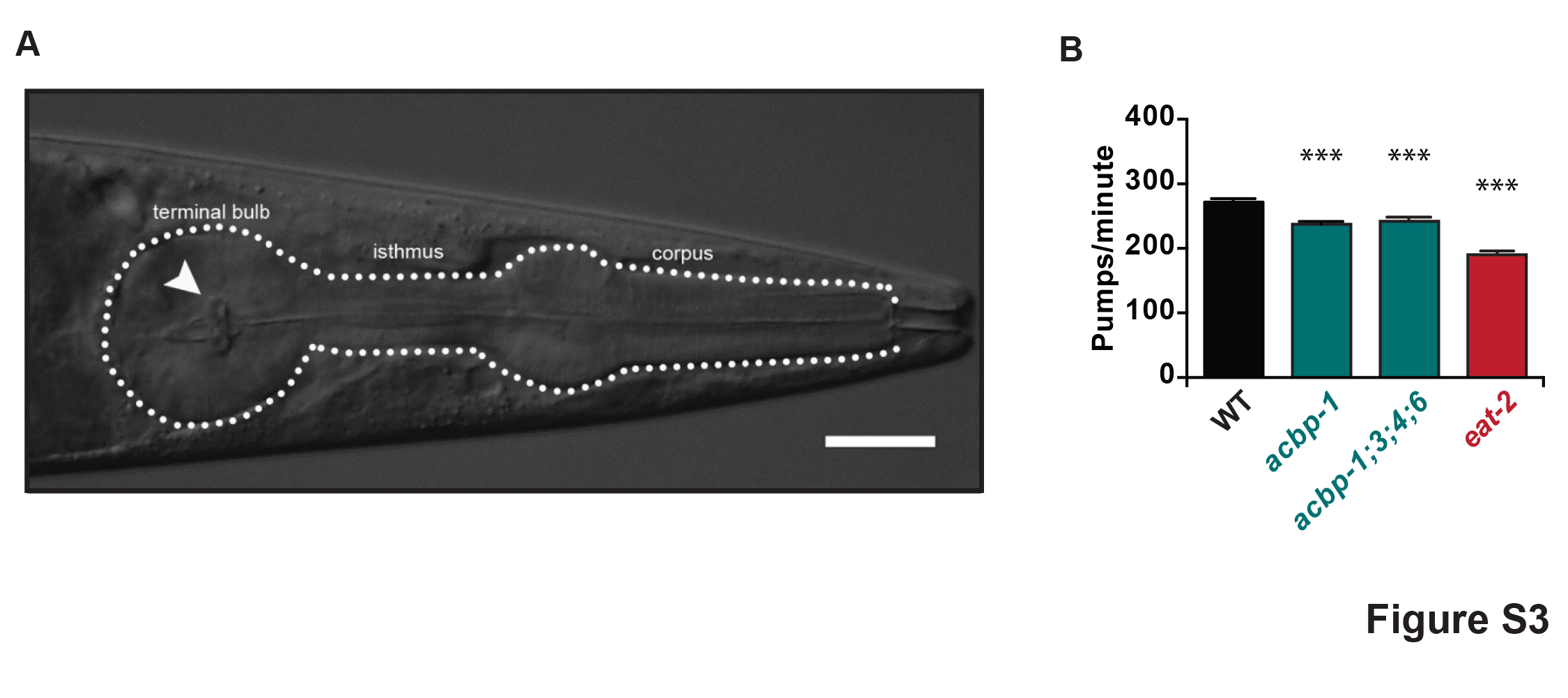

Supplement: Supplementary file 4 — Supplemental Figure S3 [file 41419_2019_2205_MOESM4_ESM.tif]

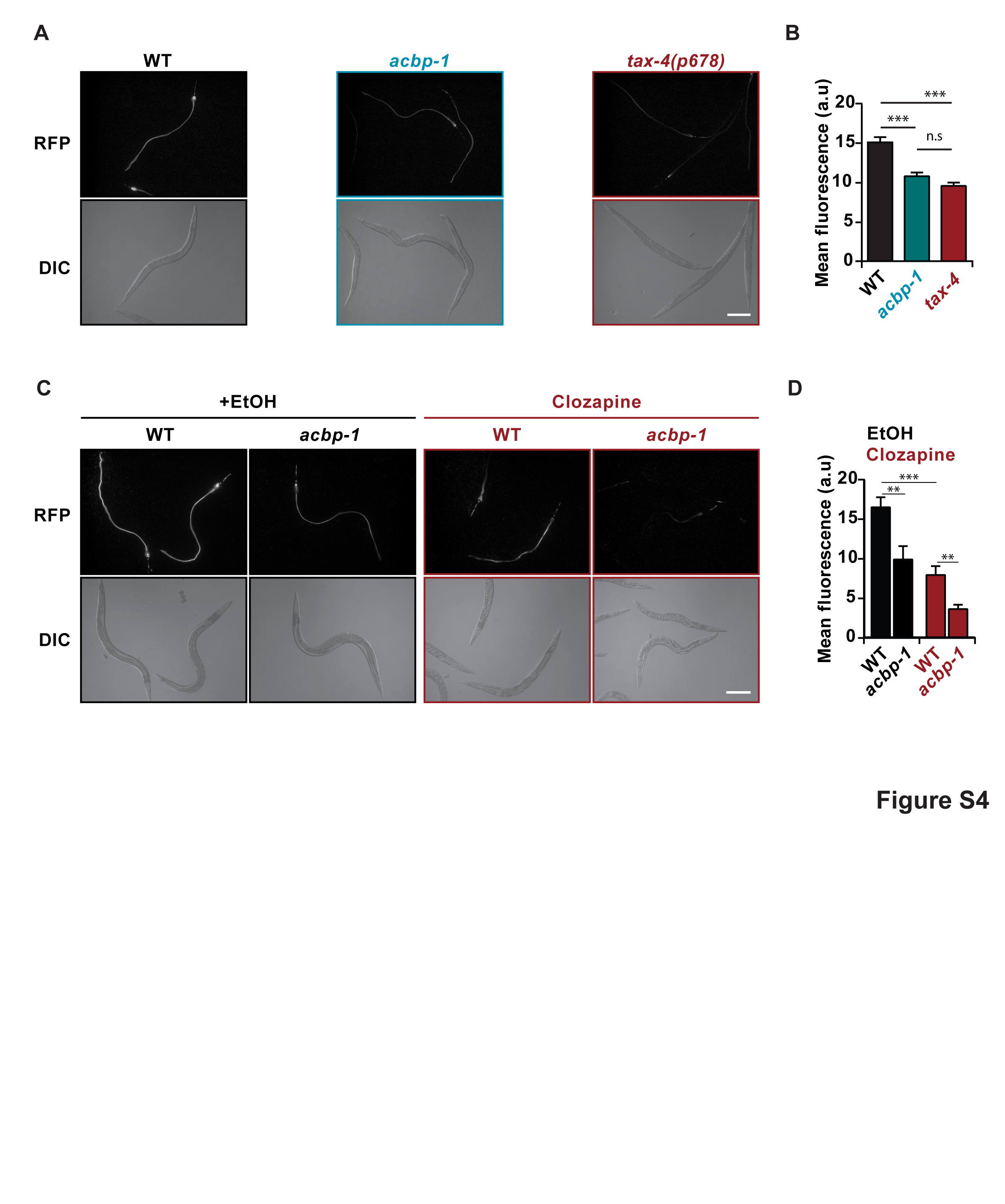

Supplement: Supplementary file 5 — Supplemental Figure S4 [file 41419_2019_2205_MOESM5_ESM.tif]
